# Associations between circulating full-length angiopoietin-like protein 8 levels and severity of coronary artery disease in Chinese non-diabetic patients: a case–control study

**DOI:** 10.1186/s12933-018-0736-6

**Published:** 2018-06-25

**Authors:** Xiaolu Jiao, Jiqiang He, Yunyun Yang, Song Yang, Juan Li, Yanwen Qin

**Affiliations:** 1 Key Laboratory of Remodeling-related Cardiovascular Diseases, Beijing An Zhen Hospital, Beijing Institute of Heart, Lung and Blood Vessel Diseases, Capital Medical University, No. 2 Anzhen Road, Chaoyang District, Beijing, 100029 China; 2 Department of Cardiology, Beijing An Zhen Hospital, Capital Medical University, Beijing, 100029 China; 3 Key Laboratory of Upper Airway Dysfunction-related Cardiovascular Diseases, Beijing An Zhen Hospital, Beijing Institute of Heart, Lung and Blood Vessel Diseases, Capital Medical University, Beijing, 100029 China

**Keywords:** Angiopoietin-like protein 8, Coronary artery disease, Gensini score

## Abstract

**Background:**

Angiopoietin-like protein 8 (ANGPTL8), which is a novel hormone produced in liver and adipose tissue, is involved in regulating lipid metabolism. Patients with diabetes and coronary artery disease (CAD) have remarkably higher levels of ANGPTL8 than those with only diabetes. However, no studies have investigated the involvement of ANGPTL8 in CAD in Chinese non-diabetic individuals. Therefore, we investigated full-length circulating ANGPTL8 levels in patients with CAD and the association between ANGPT8 levels and severity of CAD in Chinese individuals without diabetes.

**Methods:**

We performed a case–control study in 149 Chinese non-diabetic subjects, including 80 patients with CAD and 69 controls. The Gensini stenosis scoring system was used to assess the severity of CAD. Circulating full-length ANGPTL8 levels were measured by an enzyme-linked immunosorbent assay kit. The associations between circulating full-length ANGPTL8 levels and CAD were determined by multivariate logistic regression analysis. The association between ANGPTL8 levels and Gensini scores was determined by multivariate linear regression analysis.

**Results:**

Circulating full-length ANGPTL8 levels were significantly higher in Chinese non-diabetic patients with CAD compared with controls (665.90 ± 243.49 vs 462.27 ± 151.85 pg/ml, P < 0.001). After adjusting for confounding factors, we found that circulating full-length ANGPTL8 levels were an independent risk factor for CAD (odds ratio = 2.002/100 pg ANGPTL8, 95% CI 1.430–2.803, P < 0.001) and circulating ANGPTL8 levels were positively associated with the Gensini score (β = 5.701/100 pg ANGPTL8, 95% CI 1.306–10.096, P = 0.012).

**Conclusions:**

This study shows that the circulating ANGPTL8 levels are significantly increased in patients with CAD compared with controls in Chinese non-diabetic individuals. Circulating full-length ANGPTL8 levels are an independent risk factor for CAD and they are positively associated with the severity of CAD.

*Trial registration* This study was registered in the Chinese Clinical Trial Registry (No. ChiCTR-COC-17010792)

**Electronic supplementary material:**

The online version of this article (10.1186/s12933-018-0736-6) contains supplementary material, which is available to authorized users.

## Background

Coronary artery disease (CAD) is one of the leading causes of mortality and morbidity worldwide. CAD is considered the main cause of death globally because of its high prevalence in developing countries [1]. Dyslipidemia is one of the most important factors in the pathogenesis of CAD [2, 3]. Angiopoietin-like proteins (ANGPTLs) are a group of eight proteins that share structural similarity to the members of the angiopoietin protein family. Recent studies showed that genetic and therapeutic antagonism of ANGPTLs in humans and in mice was associated with levels of lipid fractions and atherosclerotic cardiovascular disease [4, 5].

Angiopoietin-like protein 8 (ANGPTL8), also known as betatrophin [6], TD26 [7], re-feeding induced fat and liver [8], lipasin [9], and PRO1185 [10], was identified as a novel hormone, which plays a major role in lipid metabolism [11]. ANGPTL8 is a new, but atypical member of the ANGPTL family, because it lacks the C-terminal fibrinogen-like domain, but shares a common coiled-coil domain at the N-terminus with ANGPTL3 and ANGPTL4 [12]. Low frequency missense variants in the ANGPTL4 (E40K) gene protect against the risk of CAD [13] and ANGPTL3 loss-of-function mutations reduce the risk of CAD in humans [5]. ANGPTL8 can regulate the cleavage and activity of ANGPTL3. ANGPTL8 coimmunoprecipitates with the N-terminal domain of ANGPTL3 in mouse plasma and increases the appearance of N-terminal ANGPTL3 in cultured hepatocytes [11].

Patients with diabetes and CAD have remarkably higher levels of ANGPTL8 than those with only diabetes [14]. However, no studies have investigated the involvement of ANGPTL8 in atherosclerotic disease in Chinese non-diabetic individuals. Therefore, in this study, we investigated circulating full-length ANGPTL8 levels in patients with CAD and the association between ANGPT8 levels and the severity of CAD in Chinese individuals without diabetes.

## Methods

The study was designed as a case–control study. The sample size was calculated by PASS 11.0 (NCSS, LLC, Kaysville, UT, USA) using logistic regression models, with *P *= 0.9, alpha = 0.05, P_0_ = 0.5, and odds ratio (OR) = 1.74 [14]. The sample size was 138 according to the calculation. Therefore, 80 patients with CAD and 69 control subjects were recruited in this study. The study design is described in detail in Additional file 1: Figure S1. All participants gave written informed consent before enrollment. The protocol was approved by the Medicine Ethics Committee of Beijing An Zhen Hospital and adhered to the Declaration of Helsinki. This study was registered in the Chinese Clinical Trial Registry (No. ChiCTR-COC-17010792).

### Cases

All consecutive patients who underwent coronary angiography in the Department of Cardiology of Beijing An Zhen Hospital between March 2017 and July 2017 were included in this study. A total of 234 patients were eligible for the study. Individuals with a diabetes, abnormal glucose tolerance, liver disease, hepatitis, liver enzyme abnormalities, renal inadequacy, cancer, or acute infectious diseases were excluded. To reduce the possibility of undiagnosed diabetes, subjects with HbA1c levels ≥ 5.7% were also excluded in this study [15, 16]. A final total of 89 participants were enrolled. CAD was defined as ≥ 50% stenotic lesions in at least one major coronary vessel, as determined by coronary angiography [17]. According to the diagnostic standard, the participants were divided into two groups: CAD (n = 80) and non-CAD (n = 9). Diagnoses of diabetes and abnormal glucose tolerance were based on the criteria of the American Diabetes Association [16, 18]. Diabetes was defined as present if any of the following characteristics was observed: history of physician-diagnosed diabetes, use of medications or insulin for diabetes; fasting glucose levels ≥ 7 mmol/l; 2-h oral glucose tolerance test ≥ 11.1 mmol/l; or random glucose levels ≥ 11.1 mmol/l. Abnormal glucose tolerance was defined as fasting blood glucose levels ranging from 6.1 to 6.9 mmol/l [18] or a 2-h oral glucose tolerance test result from 7.8 to 11.0 mmol/l [16].

### Controls

Two groups served as controls [19]. Group 1 comprised nine patients in whom significant CAD was eventually ruled out by coronary angiography in the Department of Cardiology of Beijing An Zhen Hospital. Group 2 comprised 60 volunteers from the Health Examination Center at Beijing An Zhen Hospital between March 2017 and July 2017. A total of 561 consecutive patients were originally eligible for the study. Exclusion criteria were CAD, diabetes, abnormal glucose tolerance, liver disease, hepatitis, liver enzyme abnormalities, renal inadequacy, pregnancy, cancer or acute infectious diseases, and HbA1c levels ≥ 6.0%. In these subjects, performing coronary angiography was unethical to rule out the presence of asymptomatic CAD. Therefore, the following inclusion criteria were used [19]: no abnormal Q wave or ST-T changes on electrocardiography; a negative family history of CAD and stroke; nonsmoking status; and the absence of hypercholesterolemia, hypertriglyceridemia, diabetes mellitus, and hypertension. Based on available data from epidemiological and family studies, a cohort fulfilling these criteria is expected to have a low prevalence of asymptomatic CAD [19]. A final total of 348 participants remained. The controls were matched (1:1) to cases for sex, age, and date of blood collection according to their propensity score. Twenty subjects declined to participate.

### Gensini stenosis score

The Gensini stenosis scoring system was used to assess the severity of CAD [20] by two independent experienced observers. This system grades narrowing of the lumen as 1 for 1–25% narrowing, 2 for 26–50% narrowing, 4 for 51–75% narrowing, 8 for 76–90% narrowing, 16 for 91–99% narrowing, and 32 for total occlusion. This score was then multiplied by a factor that accounted for the importance of a lesion’s position in the coronary arterial tree. The multiplication factor for a left main stem lesion was 5. The multiplication factor was 2.5 for proximal left anterior descending artery (LAD) and proximal circumflex artery lesions, 1.5 for a mid-LAD lesion, and 1 for distal LAD, mid/distal circumflex artery, and right coronary artery lesions. The multiplication factor for any other branch was 0.5. The severity of disease was expressed as the sum of the scores for the individual lesions [21].

### Anthropometric measurements

Anthropometric determinations and blood extractions were performed on a single day. Height and weight were measured with participants wearing light indoor clothing and barefoot using calibrated portable electronic weighing scales and portable inflexible height measuring bars. Blood pressure was measured after a 5-min rest in the sitting position. Blood pressure was determined at least three times at the right upper arm, and the mean value was used in the analyses. Body mass index (BMI) was calculated using the standard BMI formula: body mass (in kg) divided by square of height (in m^2^). Nonsmokers were patients who had never smoked or had stopped smoking within ≥ 1 year before enrollment in the study. All remaining patients were classified as smokers. Drinkers were defined as daily alcohol intake ≥ three times a week.

### Blood sample preparation

All blood samples were collected after the participants had fasted overnight. Blood samples were then centrifuged for 10 min at 3000 rpm and 4 °C. Plasma samples were subsequently stored in a freezer at − 80 °C before analysis. Serum triglyceride (TG), total cholesterol (TC), low-density lipoprotein cholesterol (LDL-C), and high-density lipoprotein cholesterol (HDL-C) levels, and other routine serum biochemical parameters were measured in a biochemical analyzer (Hitachi-7600, Tokyo, Japan) using blinded quality control specimens in the Department of the Biochemical Laboratory at Beijing An Zhen Hospital. Serum non-HDL-C levels were calculated by subtracting HDL-C from TC levels according to the 2016 European Society of Cardiology and European Atherosclerosis Society Guidelines for the Management of Dyslipidemias [3]. Circulating full-length ANGPTL8 levels were measured using an enzyme-linked immunosorbent assay kit (Wuhan ELAAB Science, Wuhan, China; Catalogue No. 11644h) according to the manufacturer’s instructions. Intra- and interassay coefficients of variation for ANGPTL8 levels were less than 5 and 10%, respectively.

### Statistical analysis

Continuous variables are expressed as mean ± standard deviation and categorical variables as numerals (percentages). The independent Student’s *t* test for normal distribution and the Wilcoxon rank sum test for asymmetric distribution were used to analyze the differences in continuous variables. The Chi square test was used to analyze categorical variables. The association between circulating full-length ANGPTL8 levels and CAD was determined by multivariate logistic regression analysis. The association between circulating full-length ANGPTL8 levels and Gensini scores was also evaluated using multivariable liner regression analysis. A P value of < 0.05 was considered statistically significant. Statistical analysis was performed with SPSS 20.0 (IBM Corp., Armonk, NY, USA).

## Results

### Baseline clinical characteristics of the study population

**Fig. 1:**
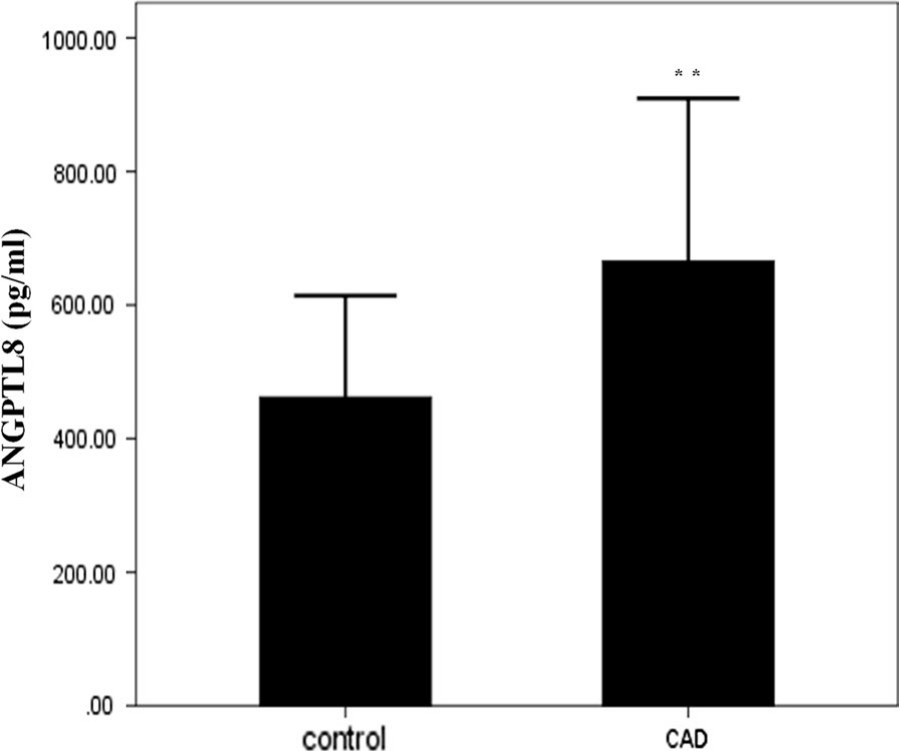
Circulating full-length ANGPTL8 levels were higher in patients with CAD compared with controls (CAD [665.90 ± 243.49] vs controls [462.27 ± 151.85] pg/ml, P < 0.001**). *ANGPTL8* angiopoietin-like protein 8, *CAD* coronary artery disease

The present study included 80 patients with CAD and 69 control subjects. The clinical characteristics of the individuals are shown in Table 1. There were no differences in fasting blood glucose (P = 0.560), TC (P = 0.927), LDL-C (P = 0.394), and non-HDL-C (P = 0.131) levels between the two groups. Patients with CAD had a significantly higher BMI compared with the control group (P = 0.020). Patients with CAD also had higher systolic blood pressure (SBP) (P = 0.002), diastolic blood pressure (DBP) (P = 0.037), and TG (P < 0.001) levels, and lower HDL-C levels compared with the control group (P < 0.001). Remarkably, circulating full-length ANGPTL8 levels were significantly higher in the CAD group compared with the control group in this study (Fig. 1, 462.27 ± 151.85 vs 665.90 ± 243.49 pg/ml, P < 0.001).

**Table 1:** Anthropometric and biochemical characteristics of the subjects included in the study

	CAD N = 80	Control N = 69	P
Age (years)	57.28 ± 9.67	55.32 ± 10.82	0.077
Male (n, %)	65 (81.25%)	53 (76.81%)	0.321
BMI	25.75 ± 2.79	24.72 ± 3.36	0.020*
Smoker (n, %)	42 (52.5%)	7 (10.14%)	< 0.001**
Drinker (n, %)	21 (26.25%)	26 (37.68%)	0.156
SBP (mmHg)	129.16 ± 15.29	120.90 ± 14.77	< 0.002
DBP (mmHg)	78.16 ± 8.66	75.19 ± 11.15	0.037
FBG (mmol/l)	5.37 ± 0.62	5.32 ± 0.44	0.317
TG (mmol/l)	1.57 (1.10–2.26)	1.04 (0.68–1.43)	< 0.001**
TC (mmol/l)	4.65 ± 1.36	4.37 ± 0.60	0.927
LDL-C (mmol/l)	2.92 ± 1.17	2.55 ± 0.52	0.394
HDL-C (mmol/l)	1.04 ± 0.23	1.26 ± 0.29	< 0.001**
Non-HDL-C (mmol/l)	3.60 ± 1.29	3.11 ± 0.63	0.131
UA (μmol/l)	336.23 ± 80.97	320.32 ± 72.17	0.258
CR (μmol/l)	76.51 ± 13.84	71.23 ± 13.74	0.028*
ALT (U/l)	25.45 ± 11.66	20.42 ± 8.75	< 0.003*
AST (U/l)	23.83 ± 8.18	21.32 ± 4.72	0.067
γ-GT (U/l)	30.74 ± 19.45	25.97 ± 14.31	0.107

Results are expressed as mean ± standard deviation, median (interquartile range) or n (%). Differences between groups were analyzed by the independent Student t test, χ^2^ text, or Wilcoxon test

*CAD* coronary artery disease, *BMI* body mass index, *SBP* systolic blood pressure, *DBP* diastolic blood pressure, *FPG* fasting plasma glucose, *TG* triglycerides, *TC* total cholesterol, *LDL-C* low-density lipoprotein cholesterol, *HDL-C* high-density lipoprotein cholesterol, *UA* uric acid, *CR* creatinine, *AST* aspartate aminotransferase, *ALT* alanine aminotransferase, *γ-GT* γ-glutamyltransferase

* P < 0.05, ** P < 0.001

### Association between circulating full-length ANGPTL8 levels and CAD

We used ordinal logistic regression analysis to estimate associations between CAD and clinical or biochemical variables. As shown in Table 2, higher BMI (OR = 1.118, 95% confidence interval [CI] 1.003–1.246, P = 0.044), SBP (OR = 1.040, 95% CI 1.015–1.066, P = 0.002), TG levels (OR = 2.048, 95% CI 1.292–3.248, P = 0.002), LDL-C levels (OR = 1.570, 95% CI 1.075–2.291, P = 0.020), and non-HDL-C levels (OR = 1.612, 95% CI 1.146–2.267, P = 0.006) were risk factors for CAD. However, HDL-C levels were a protective factor for CAD (OR = 0.037, 95% CI 0.009–0.162, P < 0.001).

**Table 2:** Associations between clinical or biochemical variables and CAD

	OR	95% CI	P value
Age (year)	1.019	0.987–1.052	0.245
Gender (male = 1, female = 2)	0.764	0.346–1.688	0.506
BMI (kg/m^2^)	1.118	1.003–1.246	0.044*
SBP (mmHg)	1.040	1.015–1.066	0.002*
DBP (mmHg)	1.031	0.997–1.067	0.072
TG (mmol/l)	2.048	1.292–3.248	0.002*
TC (mmol/l)	1.274	0.934–1.737	0.126
LDL-C (mmol/l)	1.570	1.075–2.291	0.020*
HDL-C (mmol/l)	0.037	0.009–0.162	< 0.001**
Non-HDL-C (mmol/l)	1.612	1.146–2.267	0.006*

Dependent variable: CAD

*OR* odds ratio, *CI* confidence interval, *CAD* coronary artery disease, *BMI* body mass index, *SBP* systolic blood pressure, *DBP* diastolic blood pressure, *TG* triglycerides, *TC* total cholesterol, *LDL-C* low-density lipoprotein cholesterol, *HDL-C* high-density lipoprotein cholesterol

* P < 0.05, ** P < 0.001

The association between CAD and circulating full-length ANGPTL8 levels was also tested in different models of logistic regression. Patients who had higher circulating full-length ANGPTL8 levels had a higher OR (OR = 1.709/100 pg ANGPTL8, 95% CI 1.377–2.121, P < 0.001, Table 3). After adjustment for conventional CAD risk factors, including age, sex, BMI, smoking habit, SBP, DBP, TG, TC, LDL-C, HDL-C, and non-HDL-C, increased circulating full-length ANGPTL8 levels conferred a higher OR of CAD (OR = 1.981/100 pg ANGPTL8, 95% CI 1.446–2.713, P < 0.001). We then also adjusted the levels of plasma alanine aminotransferase (ALT) and creatinine. We found that circulating full-length ANGPTL8 levels were an independent risk factor for CAD (OR = 2.002/100 pg ANGPTL8, 95% CI 1.430–2.803, P < 0.001).

**Table 3:** Multivariate logistic regression analyses of circulating full-length ANGPTL8 levels and CAD

	Unadjusted		Model 1		Model 2		Model 3	
	OR (95% CI)	P value	OR (95% CI)	P value	OR (95% CI)	P value	OR (95% CI)	P value
ANGPTL8 (per 100 pg increase)	1.709 (1.377–2.121)	< 0.001**	1.963 (1.480–2.603)	< 0.001**	1.981 (1.446–2.713)	< 0.001**	2.002 (1.430–2.803)	< 0.001**

Model 1: adjusted for age, sex, BMI, and smoker

Model 2: adjusted for Model 1 + SBP, DBP, TG, TC, LDL-C, HDL-C, and non-HDL-C

Model 3: adjusted for Model 2 + ALT and CR

*ANGPTL8* angiopoietin-like protein 8, *CAD* coronary artery disease, *BMI* body mass index, *SBP* systolic blood pressure, *DBP* diastolic blood pressure, *TG* triglycerides, *TC* total cholesterol, *LDL-C* low-density lipoprotein cholesterol, *HDL-C* high-density lipoprotein cholesterol, *ALT* alanine aminotransferase, *CR* creatinine

* P < 0.05, ** P < 0.001

Multivariate linear regression analysis was used to examine the association of circulating full-length ANGPTL8 levels and the Gensini score. After adjustment for age, sex, BMI, smoking habit, SBP, DBP, TG, TC, LDL-C, HDL-C, non-HDL-C, ALT, and creatinine, circulating full-length ANGPTL8 levels were positively associated with the Gensini score (β = 5.701/100 pg ANGPTL8, 95% CI 1.306–10.096, P = 0.012, Table 4), which represents the severity of CAD in this study.

**Table 4:** Multivariate linear regression analyses of circulating full-length ANGPTL8 levels and the Gensini score

	Unadjusted		Model 1		Model 2		Model 3	
	Β (95% CI)	P value	Β (95% CI)	P value	Β (95% CI)	P value	Β (95% CI)	P value
ANGPTL8 (per 100 pg increase)	4.641 (1.221–8.060)	0.008*	5.142 (1.300–8.984)	0.009*	5.648 (1.754–9.541)	0.005*	5.701 (1.306–10.096)	0.012*

Model 1: adjusted for age, sex, BMI, and smoker

Model 2: adjusted for Model 1 + SBP, DBP, TG, TC, LDL-C, HDL-C, and non-HDL-C

Model 3: adjusted for Model 2 + ALT and CR

*ANGPTL8* angiopoietin-like protein 8, *CAD* coronary artery disease, *BMI* body mass index, *SBP* systolic blood pressure, *DBP* diastolic blood pressure, *TG* triglycerides, *TC* total cholesterol, *LDL*-*C* low-density lipoprotein cholesterol, *HDL-C* high-density lipoprotein cholesterol, *ALT* alanine aminotransferase, *CR* creatinine

* P < 0.05, ** P < 0.001

## Discussion

In this study, we found that circulating full-length ANGPTL8 levels in patients with CAD were significantly elevated compared with controls in Chinese non-diabetic individuals. Circulating full-length ANGPTL8 levels were positively associated with the severity of CAD after adjusting for confounding factors. Circulating full-length ANGPTL8 levels were an independent risk factor for CAD.

Dyslipidemia is one of the most important factors in the pathogenesis of CAD, and ANGPTL8 plays an important role in lipid metabolism. ANGPTL8 levels are significantly and positively related to TG and LDL-C levels, but inversely related to HDL-C levels in children and patients with diabetes [22–24]. ANGPTL8 levels are also positively correlated with hepatocellular lipid content [25] and ANGPTL8 antisense oligonucleotide prevents hepatic steatosis [26]. A lot of evidence from animal studies also suggests that ANGPTL8 plays a significant role in lipid metabolism [8, 27]. Previous studies have shown that serum TG levels of ANGPTL8 null mice were one-third of wild-type. Furthermore, knockdown of ANGPTL8 during 3T3-L1 adipogenesis caused approximately a 35% decrease in TG content, but the activity of lipoprotein lipase (LPL), which is an enzyme that hydrolyzes TG circulating in capillaries of adipose tissues and muscle, was increased [8, 28]. Another study showed that adenoviral ANGPTL8 overexpression in mice increased serum TG levels, while recombinant ANGPTL8 inhibited LPL activity [29]. ANGPTL3, 4, and 8 show a sequence that binds to LPL, and ANGPTL8 requires ANGPTL3 for its effects on LPL [30]. Co-expression of ANGPTL3 and ANGPTL8 in mice results in a reduction in circulating ANGPTL3 and an increase in plasma TG levels, whereas plasma TG levels do not change with expression of ANGPTL8 alone [11]. A previous study demonstrated that ANGPTL3 was specifically correlated with HDL-C, apolipoprotein A-I, and HDL function in female non-diabetic participants [18]. Inhibition of ANGPTL8 in mice using a monoclonal antibody decreased plasma TG levels and increased LPL activity [30]. Therefore, ANGPTL8 might play a role in serum lipid metabolism either directly or indirectly (by promoting the cleavage of serum ANGPTL3). However, in our study, there was no association between serum lipids and circulating full-length ANGPTL8 levels. This may be because all of the subjects who were included in the CAD group were taking lipid-lowering drugs (statins or ezetimibe, Additional file 2: Table S1) according to the American College of Cardiology/American Heart Association cardiovascular prevention guidelines. This led to a significant reduction in serum lipid levels in this group.

ANGPTL8 has similar functions to ANGPTL3 and ANGPTL4 because the N-terminal domains of ANGPTL8 share 20% sequence identity with those of ANGPTL3 and ANGPTL4 [10]. A previous study reported that ANGPTL3 deficiency reduced the risk of coronary heart disease in humans [5]. A human monoclonal antibody against ANGPTL3 resulted in a greater decrease in atherosclerotic lesion area and necrotic content compared with a control antibody in dyslipidemic mice [4]. Serum ANGPTL4 and ANGPTL8 levels are increased in patients with hypertension [31]. ANGPTL4 missense variants (E40K) protect against the risk of CAD [13]. Genetic knockout of ANGPTL4 protects APOE^−/−^ mice against development of atherosclerosis and strongly suppresses the ability of the macrophages to become foam cells [32]. The human monoclonal antibody against ANGPTL3, named evinacumab, has been approved by the Food and Drug Administration as a new drug for treating familial hypercholesterolemia. ANGPTL3 is a new therapeutic target for atherosclerosis [33] and inhibition of ANGPTL3 can reduce the residual cardiovascular risk [34]. ANGPTL8 may activate ANGPTL3 [11]. ANGPTL8 coimmunoprecipitated with the N-terminal domain of ANGPTL3 in mouse plasma, and increased the appearance of N-terminal ANGPTL3 in cultured hepatocytes [11]. Therefore, ANGPTL8 may be a new therapeutic target for atherosclerotic cardiovascular disease. However, further research is required to determine this possibility.

Serum ANGPTL8 levels are affected by various factors, such as ethnicity and genetic and metabolic status. Lower plasma HDL-C and LDL-C levels are associated with a variant in ANGPTL8 (rs2278426, R59W) in African Americans and Hispanics, but this association is not apparent in European Americans [11]. In a Chinese Han population, people with the ANGPTL8 rs2278426 (GA/AA) genotype have lower TC and LDL-C levels than those with the GG genotype. However, there were no differences in serum lipid levels identified between the specific genotypes of ANGPTL8 in a Chinese Mulao population [35]. Fasting inhibits ANGPTL8 expression, and refeeding can highly induce its expression [11]. Elevated ANGPTL8 levels are associated with cardiometabolic risk factors, such as TC, TG, and LDL-C, but this association is largely dependent on vitamin D status [36].

Our study showed that circulating ANGPTL8 levels were positively correlated with age (Additional file 3: Table S2), which is consistent with the conclusions reached by Hu et al. [37] and Abu-Farha et al. [38]. Epidemiological evidence indicates that blood lipid levels increase with increasing age [39], which could be the reason for the elevation in ANGPTL8 levels in our study. Our study also showed that there was no association between plasma ANGPTL8 levels and BMI (Additional file 3: Table S2), consistent with a study by Roth et al. [40]. However, results regarding the association between plasma ANGPTL8 levels and BMI have been inconsistent. Some studies have indicated that ANGPTL8 levels tend to be negatively correlated with BMI [41] while others observed that plasma ANGPTL8 concentrations positively correlated with BMI [41]. To avoid a confounding effect, we adjusted for age and BMI in this study. It is reported that circulating ANGPTL8 levels is increased in patients with polycystic ovary syndrome, partly because of the levels of sex hormone [42]. In this study, we found that circulating ANGPTL8 levels is higher in men compared with women in the control group (492.73 ± 148.42 vs 361.38 ± 118.44 pg/ml, P = 0.002), while there were no differences in the levels of circulating ANGPTL8 between the two genders in patients with CAD (667.51 ± 238.24 vs 658.93 ± 273.90 pg/ml, P = 0.912). In order to avoid a confounding effect, we also adjusted for sex in this study. A previous study showed that serum ALT levels are positively associated with ANGPTL8 levels [43], while serum ALT levels were higher in CAD compared with controls in our study (Table 1). Therefore, ALT was adjusted in this study. We also found that serum creatinine was positively associated with ANGPTL8 in this study (Additional file 3: Table S2). Renal function is independently associated with circulating ANGPTL8 and circulating ANGPTL8 levels are positively correlated with serum creatinine levels [44]. However, the mechanism for the association between serum creatinine and ANGPTL8 levels is unknown. We adjusted for serum creatinine levels in this study.

Care was taken to avoid bias in this study. An enzyme-linked immunosorbent assay was performed according to the manufacturer’s instructions by a trained experimenter who was unaware of patients’ clinical data. Moreover, in statistical analysis, adjustments were made for the confounding effects of risk factors for CAD and circulating ANGPTL8 levels. Finally, propensity score matching was used to reduce the effects of outcome-selection bias.

This study has some limitations. First, it was a case–control study, which meant that it could only show associations, not causality. Second, all of the patients with CAD and some controls were taking drugs (Additional file 2: Table S1). The effects of medication on ANGPTL8 levels were not observed in this study. Third, because all of the study participants were Chinese, the findings may not be generalizable to other ethnicities. Our findings should be confirmed in other populations.

## Conclusions

Circulating full-length ANGPTL8 levels are significantly higher in Chinese non-diabetic patients with CAD compared with controls. Circulating full-length ANGPTL8 levels are an independent risk factor for CAD and are positively associated with the severity of CAD.

## Additional files

**Additional file 1: Figure S1.** Flow chart of inclusion of cases and controls in this study. Abbreviations: CAD, coronary artery disease; OGTT, oral glucose tolerance test; FBG, fasting blood glucose.

**Additional file 2: Table S1.** List of medications for participants with coronary artery disease.

**Additional file 3: Table S2.** Correlations between clinical variables and circulating full-length ANGPTL8 levels.

## Abbreviations

CAD: coronary artery disease
ANGPTL8: angiopoietin-like protein 8
TC: total cholesterol
LDL-C: low-density lipoprotein cholesterol
TG: triglycerides
HDL-C: high-density lipoprotein cholesterol
BMI: body mass index
SBP: systolic blood pressure
DBP: diastolic blood pressure
