# Supplementary figures and images for: Associations between circulating full-length angiopoietin-like protein 8 levels and severity of coronary artery disease in Chinese non-diabetic patients: a case–control study
Source: Cardiovasc Diabetol. 2018 Jun 25;17:92. doi: 10.1186/s12933-018-0736-6 (PMC6016144; doi:10.1186/s12933-018-0736-6)

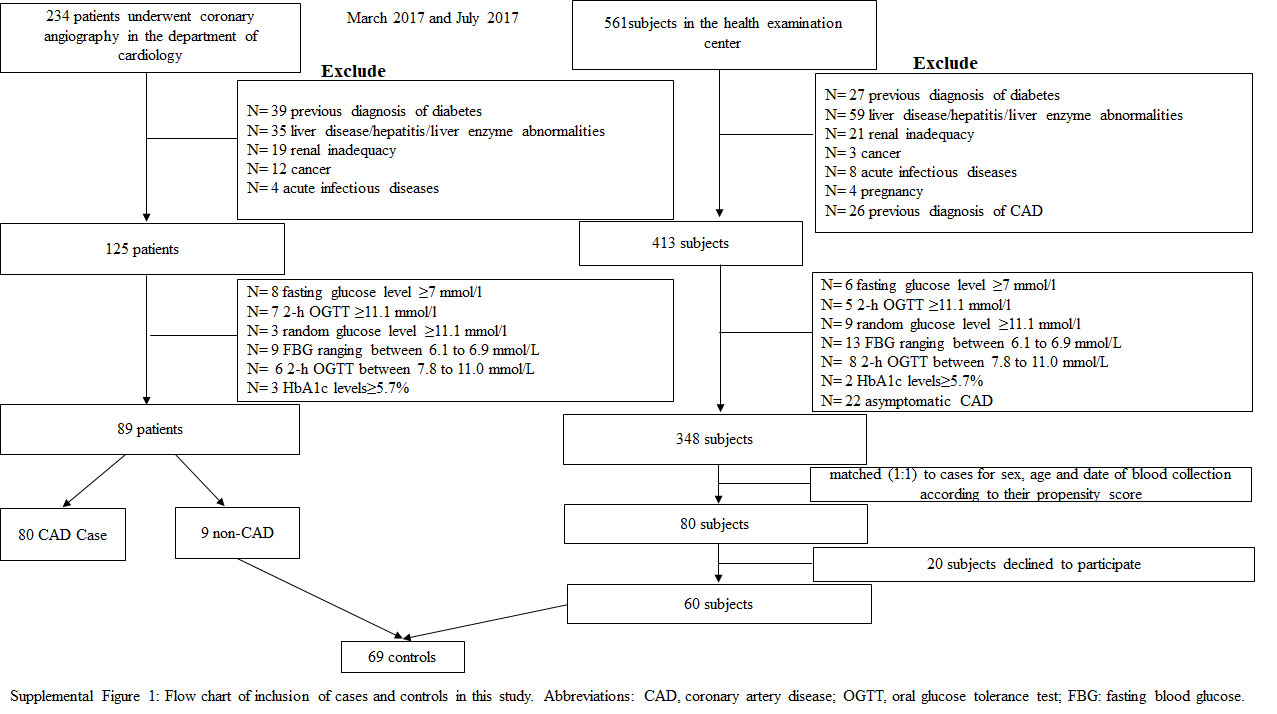

Supplement: Supplementary file 1 — Additional file 1: Figure S1. Flow chart of inclusion of cases and controls in this study. Abbreviations: CAD, coronary artery disease; OGTT, oral glucose tolerance test; FBG, fasting blood glucose. [file 12933_2018_736_MOESM1_ESM.jpg]
