# Supplementary material for: Associations between circulating full-length angiopoietin-like protein 8 levels and severity of coronary artery disease in Chinese non-diabetic patients: a case–control study
Source: Cardiovasc Diabetol. 2018 Jun 25;17:92. doi: 10.1186/s12933-018-0736-6 (PMC6016144; doi:10.1186/s12933-018-0736-6)
Supplement: Supplementary file 2 — Additional file 2: Table S1. List of medications for participants with coronary artery disease. [file 12933_2018_736_MOESM2_ESM.docx]

Supplemental Table 1. List of medications for participants with coronary artery disease.

| medication | CAD | Control |
| --- | --- | --- |
| Statins | 75 | 6 |
| Ezetimibe | 20 | 0 |
| β-blocker | 38 | 1 |
| Aspirin | 72 | 6 |
| Clopidogrel | 69 | 3 |
| Nitrates | 46 | 0 |
| Others | 42 | 5 |
