# Supplementary material for: Associations between circulating full-length angiopoietin-like protein 8 levels and severity of coronary artery disease in Chinese non-diabetic patients: a case–control study
Source: Cardiovasc Diabetol. 2018 Jun 25;17:92. doi: 10.1186/s12933-018-0736-6 (PMC6016144; doi:10.1186/s12933-018-0736-6)
Supplement: Supplementary file 3 — Additional file 3: Table S2. Correlations between clinical variables and circulating full-length ANGPTL8 levels. [file 12933_2018_736_MOESM3_ESM.docx]

Table S2. Correlations between clinical variables and circulating full-length ANGPTL8 levels.

|  | All subjects (n=149) | | |
| --- | --- | --- | --- |
|  | ρ | P-value | |
| Age (years) | 0.399 | <0.001** | |
| BMI (kg/m^2^) | -0.052 | 0.528 | |
| SBP (mmHg) | 0.223 | 0.006* | |
| DBP(mmHg) | 0.024 | 0.771 | |
| FBG (mmol/L) | 0.046 | 0.578 | |
| TG (mmol/L) | 0.053 | 0.522 | |
| TC (mmol/L) | 0.017 | 0.827 | |
| LDL-C (mmol/L) | 0.018 | 0.827 | |
| HDL-C (mmol/L) | -0.099 | 0.229 | |
| Non-HDL-C (mmol/L) | 0.025 | 0.759 | |
| UA (umol/L) | 0.103 | 0.209 | |
| CR (umol/L) | 0.344 | <0.001** | |
| ALT (U/L) | -0.079 | 0.340 | |
| AST (U/L) | 0.083 | 0.312 | |
| γ-GT (U/L) | 0.078 | 0.347 | |
|  |  |  |  |

Abbreviations: ANGPTL8, angiopoietin-like protein 8; BMI, body mass index; SBP, systolic blood pressure; DBP, diastolic blood pressure; FPG, fasting plasma glucose; TG, triglycerides; TC, total cholesterol; LDL-C, low-density lipoprotein cholesterol; HDL-C, high-density lipoprotein cholesterol; UA, uric acid; CR, creatinine; AST, aspartate aminotransferase; ALT, alanine aminotransferase; γ-GT, γ-glutamyltransferase.

*P<0.05, **P<0.001.
